# Exploring barriers to vaccine delivery in adult migrants: a qualitative study in primary care: Sally Hargreaves

**DOI:** 10.1093/eurpub/ckac130.166

**Published:** 2022-10-25

**Authors:** S Hargreaves, J Carter, A Mehrotra, F Knights, A Deal, AF Crawshaw, F Wurie, Y Ciftci, A Majeed

**Affiliations:** 1 Institute for Infection and Immunity, St George's University of London, London, UK; 2 Office for Improvements and Disparities, UK Health Security Agency London, London, UK; 3 Doctors of the World UK, London, UK; 4 Department of Primary Care and Public Health, Imperial College London, London, UK

## Abstract

**Background:**

The COVID-19 pandemic has highlighted shortfalls in the delivery of vaccine programmes to older migrant groups. Guidelines exist, however, little is known around care pathways and engagement of these older cohorts in routine vaccinations in primary care, including catch-up programmes. We explored the views of primary care professionals around barriers and facilitators to catch-up vaccination in adult migrants (defined as foreign born; 18+ years) with incomplete or uncertain vaccination status.

**Methods:**

We did a qualitative interview study with purposive sampling and thematic analysis in UK primary care (50 practices included nationally; 1 hour qualitative interviews) with 64 primary care professionals (PCPs): 48 clinical staff including GPs, Practice Nurses and healthcare assistants (HCAs); 16 administrative staff including practice managers and receptionists (mean age 45 years; 84.4% female; a range of ethnicities).

**Results:**

Participants highlighted direct and indirect barriers to catch-up vaccines in adult migrants who may have missed vaccines as children, missed boosters, and not be aligned with the UK's vaccine schedule, from both a personal and service-delivery level, with themes including: lack of training and knowledge of guidance around catch-up vaccination among staff; unclear or incomplete vaccine records; and lack of incentivization (including financial reimbursement) and dedicated time and care pathways. Adult migrants were reported as being excluded from many vaccination initiatives, most of which focus exclusively on children. PCPs noted that migrants expressed to them a range of views around vaccines, from positivity to uncertainty, to refusal.

**Conclusions:**

Vaccine uptake in adult migrants could be improved through implementing new financial incentives, strengthening care pathways and training, and working directly with local community groups to improve understanding around the benefits of vaccination at all ages.

**Key messages:**

- There are direct and indirect barriers to catch-up vaccines in adult migrants who may have missed vaccines as children, missed boosters, and not be aligned with the UK’s vaccine schedule.
- Primary care teams have a key role to play in implementing WHO’s new Immunization Agenda and to better consider catch-up vaccination to under-immunised groups across the life-course.

